# Excitation Intensity Dependent Carrier Dynamics of Chalcogen Heteroatoms in Medium-Bandgap Polymer Solar Cells

**DOI:** 10.1038/s41598-017-00834-0

**Published:** 2017-04-11

**Authors:** Chandramouli Kulshreshtha, Jiwon Son, Torbjörn Pascher, Ji-Hee Kim, Taiha Joo, Jaewon Lee, Mun Seok Jeong, Kilwon Cho

**Affiliations:** 1 Department of Chemical Engineering, Pohang University of Science and Technology, Pohang, 37673 Korea; 2 Department of Chemistry, Pohang University of Science and Technology, Pohang, 37673 Korea; 3 Chemical Physics, Kemicentrum, Lund University, SE-22100 Lund, Sweden; 4 Department of Energy Science, Sungkyunkwan University, Suwon, Korea

## Abstract

The excitation intensity dependent carrier dynamics of blends with PC[70]BM of three new medium-band gap conjugated polymers with central chalcogen heteroatoms, PBDTfDTBX (X = O, T(Sulphur), Se) were studied. The PBDTfDTBX polymers (Poly[4,8-bis(5-(2-butyloctyl)thiophene-2-yl)benzo[1,2-b;4,5-b′]dithiophene-*alt*-4,7-bis(4-(2-ethylhexyl)-2-thienyl)-dithieno[3′,2′:3,4;2″,3″:5,6]benzo[1,2-c][1,2,5] furazan or thiadiazole or selenadiazole]) have symmetrical structures but exhibit different solar cell performances. In this study, we determined how the photogenerated charge carrrier dynamics of the PBDTfDTBX:PC[70]BM blends varies with the heteroatom by performing transient absorption measurements at various excitation intensities. It was found that the charge carrier dynamics of the PBDTfDTBX blends with X = T or Se heteroatoms are dependent on the excitation intensity whereas that of the PBDTfDTBO blend is independent of the intensity. The photogenerated charge carrier dynamics of the PBDTfDTBO:PCBM, PBDTfDTBT:PCBM, and PBDTfDTBSe:PCBM blends were all modeled globally and rates were estimated for different photophysical processes occurring on different time scales.

## Introduction

Organic solar cells (OSC) have attracted much attention as promising clean and renewable energy resources and provide power conversion efficiencies (PCE) that have undergone significant improvements over the last decade through the development of novel donor polymers^1–5^. The use of heterocyclic semiconductors containing chalcogens such as oxygen, sulphur, and selenium as donor polymers has rapidly spread because of their facile packing in planer structures, and because they also exhibit high carrier mobilities and stabilities. Materials containing sulphur heteroatoms are of particular interest as semiconductors for OSC applications because S- atoms can enhance intermolecular interactions. This enhancement not only facilitates charge transport but can also improve the packing motifs of organic semiconductors. Another simple method for the control of the band gaps of D–A polymers is the substitution of sulphur with other chalcogen atoms such as oxygen, selenium or tellurium^6, 7^. Thiophene-based π-conjugated systems have been extensively tested, but their oxygen- or selenium- containing counterparts have lately drawn considerable attention as novel D–A polymers that exhibit promising optoelectronic properties^8^. The variations in the polarizability and electronegativity of chalcogen atoms result in variations in the carrier dynamics of blends of chalcogen-containing polymers, and thus atomic substitutions of these fused heteroaromatic rings can be used to reveal the key parameters determining PCEs. It has been established that D–A polymers containing S heteroatoms play a dominant role in the efficiency of charge transport in OSCs but important questions about subsequent processes, such as charge separation and charge extraction, still remain. These processes compete with charge recombination which is a loss channel; therefore it is important to understand their dynamics. By varying the chalcogen atom, we have obtained insights into this dynamics that are expected to be useful to the future development of solar cell materials. Only a few reports of the carrier dynamics of blends of PCBM with polymers containing chalcogen heteroatoms in solar cell materials have been published to date; for instance no difference was found between the ultrafast decay dynamics of annealed films of P3HT (Poly-(3-hexyl)thiophene) and P3HS (Poly(3-alkyl)selenophene)^9^. Several studies on the carrier dynamics of S-containing polymeric materials for solar cell device applications^10, 11^ have been performed but there has been no comparative study on materials containing other chalcogen heteroatoms. Further, there have been no previous reports of the photogenerated excitation intensity dependence carrier dynamics of the D–A polymers with PCBM blends containing chalcogen heteroatoms in polymer solar cells.

To this end, we synthesized the new high-performance medium-band gap polymers PBDTfDTBX (X = O, T(S), Se) with chalcogenadiazole units i.e. (Poly[4,8-bis(5-(2-butyloctyl)thiophene-2-yl)benzo[1,2-b;4,5-b′]dithiophene-*alt*-4,7-bis(4-(2-ethylhexyl)-2-thienyl)-dithieno[3′,2′:3,4;2″,3″:5,6]benzo[1,2-c][1,2,5] furazan or thiadiazole or selenadiazole]), and investigated in detail their device characteristics, which have been published elsewhere^12^. In PBDTfDTBX molecule (*E*_g_ > 1.7 eV), the central atom in the polymer backbone is O, S or Se atom. The photogenerated charge carrier dynamics of blends of PBDTfDTBX (X = O, T(S), Se) with fullerene [6,6]-phenyl-C-70-butyric acid methyl ester (PC[70]BM), in which PBDTfDTBX acts as the donor and PC[70]BM acts as the acceptor, were studied by using femtosecond transient absorption pump-probe spectroscopy. The charge carrier dynamics of each of the three blends were then modeled.

**Figure 1:**
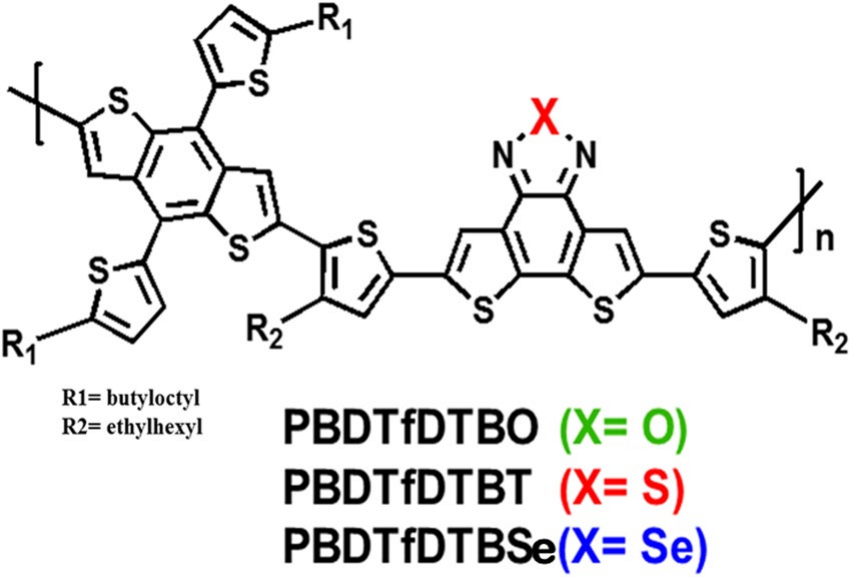
Chemical structure of polymer PBDTfDTBX (X = O, T(S), Se).

**Figure 2:**
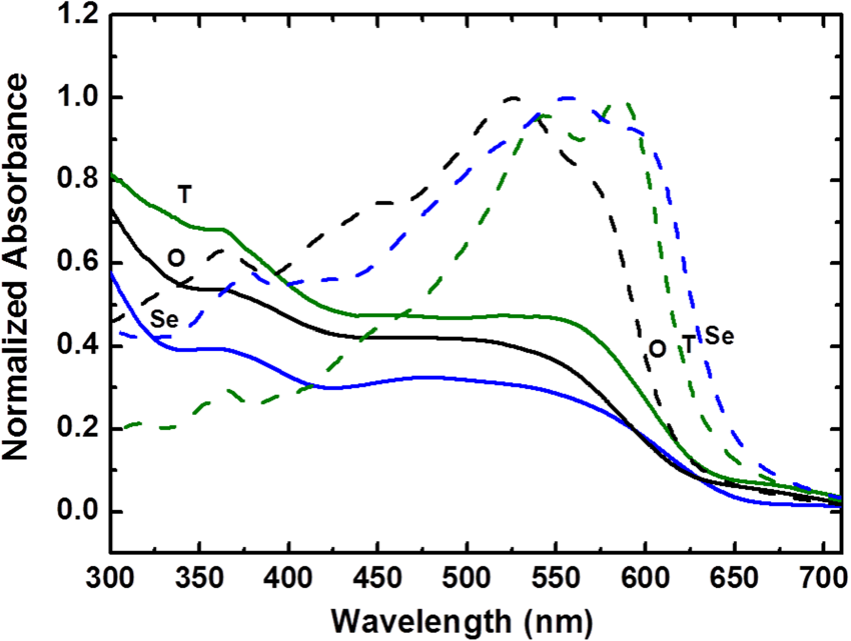
Normalized UV-Vis absorption spectra of the PBDTfDTBX (X = O, T, Se) polymers (dashed lines) and PBDTfDTBX:PC[70]BM blends (solid line). The black, blue and olive lines are the spectra for X = O, T(Sulphur), Se forms of PBDTfDTBX respectively.

The chemical structure of PBDTfDTBX (X = O, T(S), Se) is shown in Figure 1. The HOMO and LUMO energy levels and molecular weights of the polymers are given in Table SI1. The effects of varying the heteroatom on the optical properties of the polymers were investigated. The UV−VIS absorption spectra of neat polymers and their blend films were acquired in chlorobenzene solution and are shown in Figure 2. The absorption spectra of polymers contain two spectral features, a higher-energy band attributed to the localized π-π* transition and a lower-energy band ascribed to the intramolecular charge transfer (ICT) transition, as is typical of the dual-band spectra of D–A copolymer systems^13, 14^. There is a clear trend in the optical gaps of these three polymer films. The entire dual-band spectrum shifts to a lower energy as the heavier chalcogens are substituted into the polymers. The Se heteroatom is much larger and less electronegative than the O and S atoms, so Se-containing polymers are expected to be more effective in extending the absorption spectrum toward the infrared region^15^. The lower ionization potential of each heavier atom and the decrease in electronegativity down the chalcogen group leads to destabilization of the occupied bonding molecular orbital relative to the LUMO. This effect stabilizes the LUMO of the polymer, which narrows the band gap and produces a red-shift in the absorption spectrum. Furthermore, the S-containing polymer exhibits the most distinct vibronic peak than those of its analogues. The vibronic peaks of conjugated polymers are due to interchain π-π* transition, so the result indicates that the polymer chains of PBDTfDTBT undergo very strong aggregation, which is probably due to the result of higher molecular weight of PBDTfDTBT (89 kg/mol). The dependence of solar cell performance on the molecular weight (MW) of the PBDTfDTBT polymer was considered previously: a low MW was also synthesized and tested^12^: the performance difference between the low and high molecular weight devices was not too large. To explore the charge carrier dynamics of these polymers containing chalcogen heteroatom, a high MW PBDTfDTBT polymer was used as a blend with PC[70]BM. In the transient absorption measurements, we used 550 nm femtosecond pulses to excite the ground state of all three polymers in the blends.

The photovoltaic performance parameters of the devices containing PBDTfDTBO:PCBM, PBDTfDTBT:PCBM, and PBDTfDTBSe:PCBM blends are given in Table SI2. The three devices clearly differ in PCEs as shown in the J-V plots in Figure SI3: PBDTfDTBO:PCBM (1.07%), PBDTfDTBT:PCBM (3.84%), and PBDTfDTBSe:PCBM (2.32%). Devices prepared with the additive di-iodoctane exhibit slightly higher performances^12^ but our focus was the basic mechanism of the devices without any additive with polymers containing chalcogen heteroatoms. In the measurements of the transient absorption of solar cell blends, the selected blending ratios for PBDTfDTBO:PCBM (1:1), PBDTfDTBT:PCBM (1:1.5), and PBDTfDTBSe:PCBM (1:1) are similar to those that provide optimized device performance.

**Figure 3:**
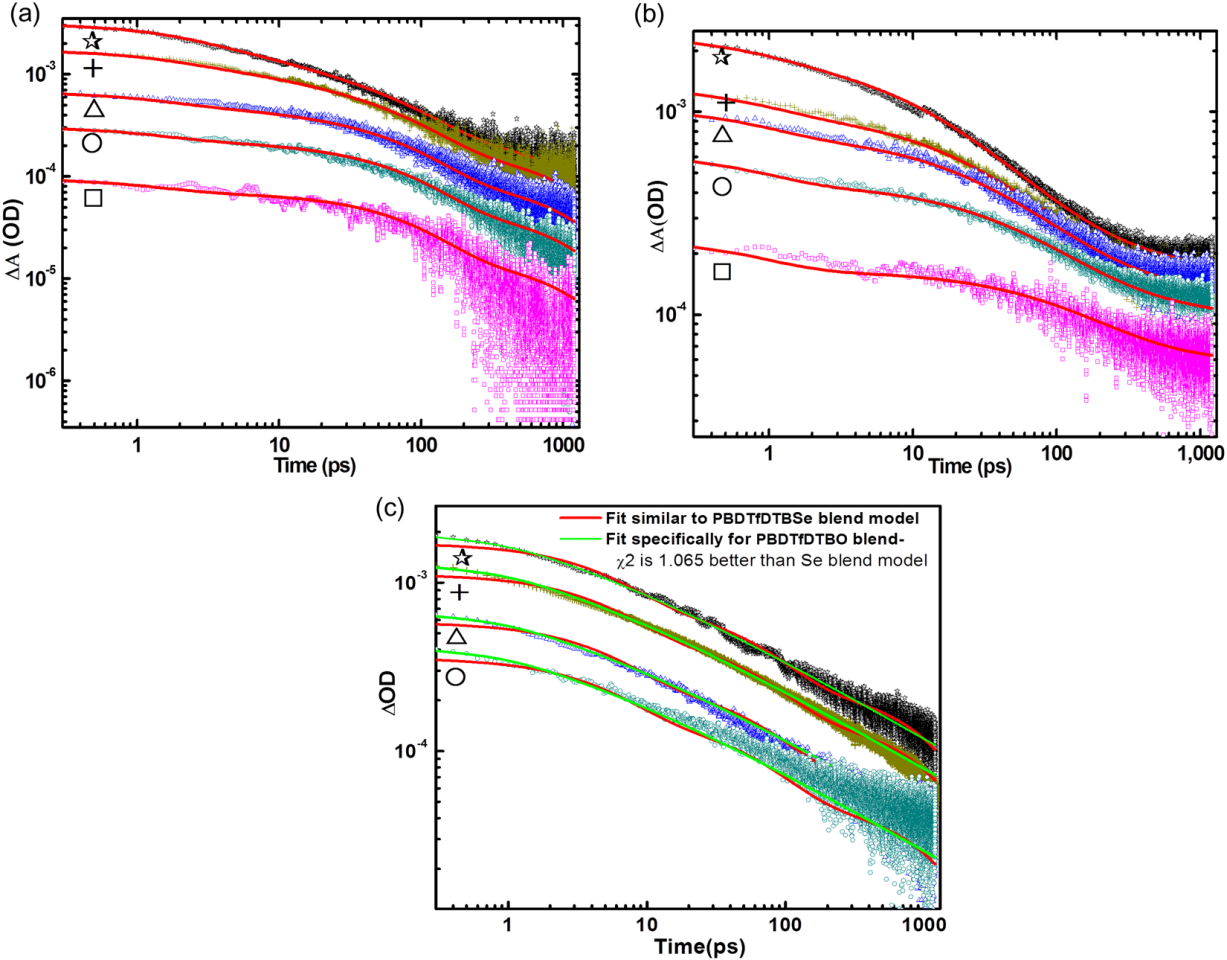
Intensity dependences of the transient absorption kinetics of the **(a)** PBDTfDTBT:PC[70]BM (1:1.5) and **(b)** PBDTfDTBSe:PC[70]BM (1:1) blends at various excitation fluence (ph/cm^2^/pulse). The fluences are color coded as follows: 2.8 × 10^14^ (black, ☆), 9.4 × 10^13^ (dark yellow, +), 5.7 × 10^13^ (blue, Δ), 2.8 × 10^13^ (dark cyan, ○), 9.4 × 10^12^ (magenta, □). **(c)** PBDTfDTBO:PC[70]BM (1:1) blend at various excitation fluence (ph/cm^2^/pulse). The fluences are color-coded as follows: 3.6 × 10^14^ (black, ☆), 1.23 × 10^14^ (dark yellow, +), 6.15 × 10^13^ (blue, Δ), 3.69 × 10^13^ (dark cyan, ○). Fitting in green is performed specifically for the results for the PBDTfDTBO:PC[70]BM (1:1) blend. The red fits shows the fitting results obtained for the PBDTfDTBO:PC[70]BM (1:1) blend with the model used for the other two blends.

The transient absorption (TA) spectra of the PBDTfDTBO:PCBM, PBDTfDTBT:PCBM, and PBDTfDTBSe:PCBM blends measured at a pump excitation of 550 nm and the highest fluence (2.8 × 10^14^ (photon/cm^2^)/pulse) over the range 1000 to 1500 nm with various delay times are shown in Figure SI1(a),(b), and (c). For all three blends, a broad excited-state absorption band peaking near 1200 nm is evident, and decayed on a picosecond time scale. The dependences on the incident light intensity of the TA kinetics of the PBDTfDTBO:PCBM, PBDTfDTBT:PCBM, and PBDTfDTBSe:PCBM blends were also examined because the carrier dynamics depend solely on the charge carrier concentrations. Figure 3(a),(b) and (c) show the intensity dependences of the kinetics for all the three blends probed at 1150 nm with the excitation intensity varied over nearly two orders of magnitude. The global fits obtained with a model derived for the PBDTfDTBSe:PCBM blend are also applicable to the PBDTfDTBT:PCBM blend, as shown in red in Figure 3(a),(b) and (c). However, this model does not satisfactorily fit the results well for the PBDTfDTBO:PCBM blend; so a special model was derived for PBDTfDTBO:PCBM blend; the global fits are shown in green in Figure 3(c). The decay measurements were performed for all three blends for 1.2 ns. We start analyzing the PBDTfDTBT:PCBM and PBDTfDTBSe:PCBM blends, since they show similar behavior. It can be seen that the kinetics of the PBDTfDTBT:PCBM and PBDTfDTBSe:PCBM blends are strongly intensity dependent up to a fluence level of 2.8 × 10^14^ (photon/cm^2^)/pulse i.e. faster decay arises as a result of nongeminate recombination, and the decay becomes slower at lower fluences i.e. 5.7 × 10^13^ (photon/cm^2^)/pulse due to the less concentration of mobile charges. The first order processes were assumed to fit the results for the lowest pump intensity. The exact nature of these slowest processes could not be determined with certainty, because we only observe the beginnings of these reactions, so we cannot completely exclude the possibility that they are nongeminate recombination process. The intensity dependences of the high intensity traces can be fitted by the addition of a time-dependent second order recombination process.

Therefore, the TA kinetics of all three blends were modeled according to the reaction scheme given in Table 1, which accounts for the charge generation, charge separation, charge recombination, geminate or nongeminate processes in the blends. The fitting of the kinetic data was performed globally, i.e. in all fluences simultaneously, according to the reaction scheme models, and the numerical values for the resulting rate constants of the reaction steps are summarized in Tables 2 and 3. This fitting model is derived from those used for APFO3 and TQ1 blends^16, 17^. By employing this model, we are able to fit the polymer excitonic decay with a single exponential with a rate constant k_CG_ for charge generation. Similarly, we used a single exponential decay for charge separation with a rate constant k_CS_. To model the intensity dependences of the PBDTfDTBT and PBDTfDTBSe blends, a second order recombination of charge pairs with a time dependent rate constant γ_1_(t) = γ_01_/(t/t_ref_)^α1^, where γ_01_ is the rate constant at a defined time t_ref_ (in this case 1 ps), and α_1_ is the change of rate with time, was used^16^. Finally, a single exponential representing a possible geminate recombination was used to model the final process evident in the S and Se traces. The geminate or nongeminate charge recombination rates of the PBDTfDTBT and PBDTfDTBSe blends with PC[70]BM were fitted reasonably well for all fluences, but considerably high recombination rate was expected in the PBDTfDTBO blend for any of the measured fluences. This effect was further observed in the normalized decays of the PBDTfDTBO blend, which overlapped even at the highest excitation intensity used in the measurements. As shown in Figure 3(c) (red), the model derived for the PBDTfDTBSe blend does not adequately describe the PBDTfDTBO blend. This problem indicates that the reaction mechanism of the PBDTfDTBO blend is completely different from others.

**Table 1:** The transition and processes, occurring within PBDTfDTBO, PBDTfDTBT, and PBDTfDTBSe blends, with their reaction orders.

Transition	Rate	Order	Process
S_0_ + hν → S_1_			photoexcitation
S_1_ → (e^−1^:h^+1^)	k_CG_	first	charge formation
(e^−1^:h^+1^) → e^−1^ + h^+1^	k_CS_	first	charge separation
(e^−1^ + h^+1^) → S_0_	k_GR_	first	geminate recombination
(e^−1^ + h^+1^) + (e^−1^ + h^+1^) → (e^−1^ + h^+1^) + S_0_	γ_1_	second	charge-pair annihilation
S_1_ → e^−1^ + h^+1^	k_CGS_	first	charge formation & separation
e^−1^ + h^+1^ + e^−1^ + h^+1^ → e^−1^ + h^+1^ + S_0_	γ_2_	second	nongeminate recombination

S_1_ are excitons generated by light absorption, (e^−1^:h^+1^) is a coulombically bound charge pair (CT state), (e^−1^ + h^+1^) is a loosely bound charge pair, e^−1^ + h^+1^ are free electron and holes.

**Table 2:** Modeling results of PBDTfDTBT:PCBM and PBDTfDTBSe:PCBM blends.

Parameters	PBDTfDTBT:PCBM	PBDTfDTBSe:PCBM
k_CG_ × 10^−12^	0.8	1.1
k_CS_ × 10^−9^	10	2.8
k_GR_ × 10^−9^	0.4	0.67
γ_01_ × 10^−12^	60	38
α_1_	0.57	0.17

Since the extinction coefficients of the species are not known, standard units for the second order rate constants could not be obtained. Instead [absorbance units/second] was used.

**Table 3:** Special model for PBDTfDTBO blend, and its reaction order.

Transition	Rate	Value	Order	Process
S_0_ + hν → S_1_				photoexcitation
S_1_ → e^−1^ + h^+1^	k_CGS_ × 10^−12^	1.6	first	charge formation & separation
(e^−1^ + h^+1^) + (e^−1^ + h^+1^) → (e^−1^ + h^+1^) + S_0_	γ_02S_ × 10^−12^	120	second	nongeminate recombination
	α_2_	0.52		
	S	0.99		

S_1_ are excitons generated by light absorption, (e^−1^ + h^+1^) are a loosely bound charge pair. Since the extinction coefficient of the species is not known, standard units for the second order rate constants could not be obtained. Instead [absorbance units/second] was used.

While comparing the end of the traces for PBDTfDTBSe blend, i.e. Figure 3(b) and the PBDTfDTBO blend i.e. Figure 3(c), the PBDTfDTBSe blend levels out and becomes almost flat in the end, but the PBDTfDTBO blend continues to decay. This decay is almost linear in the log-log plot which is indicative of a second-order recombination reaction. Note that the traces do not go together to one line as expected for a second order solution reaction, but form parallel lines. This effect has been seen for other polymers such as TQ1, P(T_0_TT_16_), and P3HT^17–19^. The proposed model for the PBDTfDTBO blend is shown in Table 3 and the resulting fits are shown in Figure 3(c) (green); the model consists of a combined charge generation/separation process with a rate constant, k_CGS_ and a time and initial concentration dependent second order recombination rate γ_2_(t).

1 $${{\rm{\gamma }}}_{2}({\rm{t}},{\rm{c}}({{\rm{n}}}_{0}))=\frac{{{\rm{\gamma }}}_{02{\rm{s}}}}{{(\frac{{\rm{c}}({{\rm{n}}}_{0})}{{{\rm{c}}}_{{\rm{ref}}}})}^{{\rm{s}}}{(\frac{{\rm{t}}}{{{\rm{t}}}_{{\rm{ref}}}})}^{{{\rm{\alpha }}}_{2}}}$$

where, ${\gamma }_{2}(t,c({n}_{0}))$ is the time and initial concentration dependent nongeminate recombination rate, t_ref_ is 1 ps, S is a scaling factor that describes how the initial concentration influences the rate constant (S = 0, no influence, S > 0, faster rate for lower fluence, S < 0 faster rate for higher fluence), c(n_0_) is the ΔOD (optical density) at t = 0 for each fluence, see Table 3 for details.Considerably better fits were obtained for PBDTfDTBO blend, which indicates that early time (<1 ns) charge carrier mobility in the PBDTfDTBO blend is significantly higher than those in the other two blends which leads to the fast second order depletion of charges resulting in poor solar cell performance. It is to be noted here that the model for PBDTfDTBSe and PBDTfDTBT blends use 8 parameters compared to 6 for the PBDTfDTBO blend. Despite this 25% reduction in parameters, the χ^2^ is 1.065 times better for the 6 parameter PBDTfDTBO blend model as compared to the 8 parameter PBDTfDTBSe model when fitting the PBDTfDTBO blend data. This clearly indicates the difference in reaction scheme for the PBDTfDTBO blend.

The charge transfer (CT) times for the PBDTfDTBT:PCBM, PBDTfDTBSe:PCBM, and PBDTfDTBO:PCBM blends are 1.3 ps (k_CG_ = 0.80 × 10^12^ s^−1^), 910 fs (k_CG_ = 1.1 × 10^12^ s^−1^), and 625 fs (k_CGS_ = 1.6 × 10^12^ s^−1^), respectively as shown in Table 2. These charge generation rates were determined by fitting the results to a single exponential decay and they confirm that charge transfer in the PBDTfDTBO:PCBM blend is faster than PBDTfDTBT:PCBM and PBDTfDTBSe:PCBM blends. Efficient charge transfer from polymer to PCBM molecules occurs in a time lesser than usual hopping time for polymers^16^. The charge transfer from polymer to fullerene molecule is considerably most effective in the PBDTfDTBO:PCBM blend. Its CT time is fastest among of the three blends. Also, the charge transfer time of the PBDTfDTBT:PCBM blend is slower than that of PBDTfDTBSe:PCBM blend, which indicates that the larger atomic size of selenium as well as its easy interaction with PCBM domains made the blend feasible for efficient charge transfer. Nevertheless, low solar cell device performance of PBDTfDTBO:PCBM than that of PBDTfDTBT:PCBM and PBDTfDTBSe:PCBM blends was of another concern.

In an operating solar cell, the bound CT states must overcome the columbic attraction and dissociate in order to provide free charge carriers or charge-separated states. Usually, charge pairs are separated over a time of ~100 ps by having a separation distance of more than 5 nm, where the coulomb interaction is effectively broken^20^. It has also been reported that driving energy for charge dissociation is provided by energy released during relaxation of CT states^21, 22^. In PBDTfDTBT:PCBM and PBDTfDTBSe:PCBM blends, charge separation times are of the order of 100 ps and 357 ps, respectively. Noted that the speed of charge separation follows the trend in the size of heteroatom, i.e. it becomes slower as the size of heteroatom increases. For instance, the selenium-containing polymeric blend generates more excitons because of its larger interaction with PCBM domains and exhibits greater exciton migration and faster electron transfer to the PCBM acceptor. Therefore, a lower columbic potential or driving energy is required for charge separation in this blend. It is also reported that charge delocalization at the donor-acceptor interface is the key parameter for the charge separation^21, 22^. The PBDTfDTBSe:PCBM blend generates more excitons which require a lower exciton splitting energy because of fast and efficient charge transfer. Therefore, the large population of charge carriers undergoes charge dissociation that slowly diffuses and reaches to the electrodes for efficient charge extraction. However, the PBDTfDTBT blend is slightly different from PBDTfDTBSe blend: there are less charge carrier recombination and efficient charge transfer, so the charge carriers undergo excitonic splitting with a low driving force and greater separation distances.

Since k_CGS_ is the charge generation and separation rate constant according to the special model for the PBDTfDTBO:PCBM blend, for which the charge generation and separation time is ~625 fs (k_CGS_ = 1.6 × 10^12^ s^−1^). This result indicates that the quenching time for the excited states in all three blends is the same within a factor of two.

In terms of charge dissociation, the sizes of the O and S heteroatoms in PBDTfDTBO and PBDTfDTBT blends are quite optimum for the delocalization of charges within the conjugated heterocycle’s π-systems, but the electronegativity of the chalcogens affects the incorporation of electrons into the heterocycle’s π-system and its dipole moment. The low electronegativity of selenium means that N−selenium bonds are polarized as N^δ−^−Se^δ+^; conversely, N−oxygen bonds are polarized as N^δ+^−O^δ−^. Therefore, the electrons on sulphur and selenium are more readily incorporated into the conjugated system than those on oxygen^23^ which results in a reduction in the activation barrier and thus promotes efficient charge separation.

Although the PBDTfDTBO:PCBM blend exhibits faster charge dissociation but it is to be noted that charge recombination also commences quite early. This means that even low concentration of charge carriers that were dissociated undergoes faster recombination.

Now coming to the charge carrier recombination, as excitation intensity decreases, the electron-hole distance increases, so the columbic interaction becomes weaker and geminate recombination can be identified. Table 2 shows the modeling results which give some insight into the amount of recombination occurring in the PBDTfDTBT:PCBM and PBDTfDTBSe:PCBM blends. The estimated rates of first order geminate recombination in the PBDTfDTBT:PCBM and PBDTfDTBSe:PCBM blends are (0.4 (ns)^−1^) and (0.67 (ns)^−1^), respectively. While observing PBDTfDTBO blend in Figure 3(c), it confirms that it has a constant linear slope in the log-log plot which is mainly indicative of a second order recombination, but the lines are separated. This means that the photogenerated carrier dynamics of the PBDTfDTBO blend is intensity independent and possibility of geminate recombination could be there but moreover this blend is dominated by second order recombination even after decreasing the population of charge carriers. Consequently, charges would recombine nongeminately that mainly competing for charge dissociation. Although, the rates for these three blends have been determined by modeling but due to limitation of time window, we see the prospects of recombination quite similar to previously studied polymer e.g. APFO3^16, 17^. For comparison, the impacts on the kinetics of PBDTfDTBO:PCBM, PBDTfDTBT:PCBM, and PBDTfDTBSe:PCBM blends of lower excitation fluence are plotted in Figure SI2(b). These normalized low fluence kinetic traces for three blends decay in the order as: PBDTfDTBT blend < PBDTfDTBSe blend < PBDTfDTBO blend, which shows that the charge recombination rates of the three blends are quite different. Further, charge recombination starts early for the PBDTfDTBO blend than for the PBDTfDTBT or PBDTfDTBSe blends. However, the PBDTfDTBT:PCBM blend possesses high molecular weight and crystallinity with efficient charge transfer because its CT states are short-lived, and hence the possibility of geminate recombination is reduced. In contrast, PBDTfDTBSe:PCBM blend also possesses good crystallinity and efficient charge transfer but the larger atomic size of selenium might produce additional trap states in its blends, which is the subject of further investigation. As mentioned above, PBDTfDTBO:PCBM blend has shown poor solar cell device performance, given in Table SI2. Its photocurrent is extremely weak because charge pairs that recombine are too strongly-bound to be dissociated and can’t be extracted out to an external circuit.

In our modeling of the intensity dependence kinetics in the PBDTfDTBO:PCBM, PBDTfDTBT:PCBM, and PBDTfDTBSe:PCBM blends, another form of second order recombination, i.e. recombination of closely coupled charge pairs or charge pair annihilation, γ_1_, was also taken into account. A small fraction of early separated charges may recombine nongeminately at high intensities. The observation of a saturation level of illumination intensity in OSC devices has been discussed in previous reports^24, 25^. It was proposed that the photocurrent increases linearly with illumination intensity whereas the open-circuit voltage at first increases linearly at low intensity of light and slowly starts saturating at high intensities of light. Thus, more charges undergo recombination in the form of charge pair annihilation at high intensities. This process occurs at early times when carrier densities are very high, which results in a reduction in the number of separated charge pairs formed: the greater the value of γ_1_, the faster is the charge pair recombination. The second order rate constant (γ_1_) of the PBDTfDTBT:PCBM blend is slightly higher than that of PBDTfDTBSe:PCBM blend, which means that charge pair recombination occurs until very late and thus inhibits the arrival of the charges to reach towards the electrodes subsequent to charge separation. Further, as mentioned above, the PBDTfDTBSe:PCBM blend has a higher charge densities because of the larger atomic size of selenium as well as its easy interaction with PCBM domains, so at high fluences there is an increase in the charge density which makes this second order recombination dominant over a longer time. We compared the highest excitation fluences of the PBDTfDTBO:PCBM, PBDTfDTBT:PCBM, and PBDTfDTBSe:PCBM blends in normalized plot in Figure SI2(a). At the highest fluence, the three blends decay in a similar fashion although the PBDTfDTBSe blend at 100 s of ps decays slightly faster than others, which confirms that its charge transport is inefficient due to higher nongeminate recombination. We believe that increasing charge densities into the blend results into more nongeminate recombination. However, the PBDTfDTBO:PCBM blend has quite different properties. In this blend, second order recombination dominates at all pump intensities leading to fast charge recombination and hence poor solar cell performance. Intensity independence is evident for all measured fluences and overall charge recombination also commences quite early, hence it is difficult to estimate the exact value of the rate for charge pair annihilation from the modeling.

**Figure 4:**
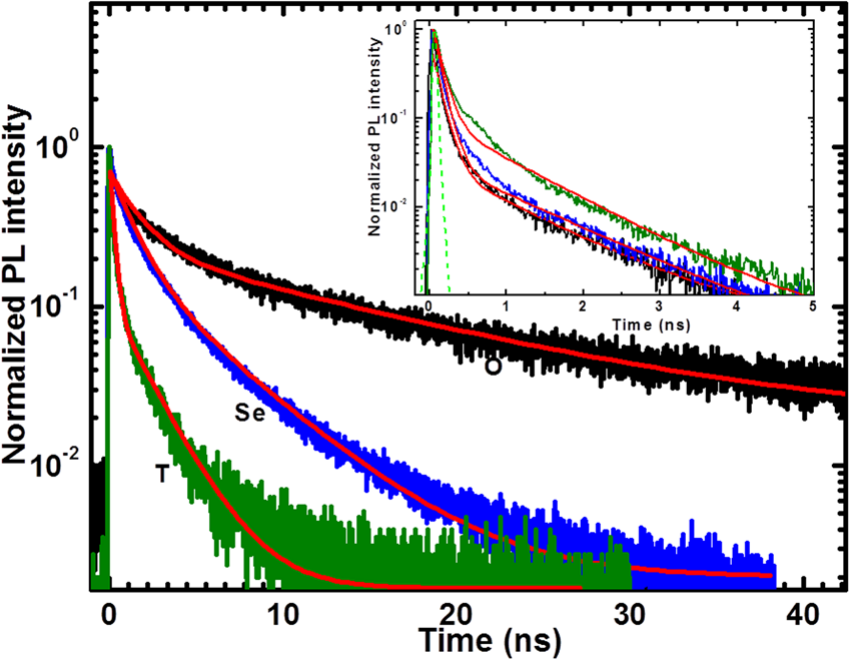
Time-correlated single photon counting fluorescence measurements for PBDTfDTBX:PC[70]BM blends, where X = O (black), T (olive) and Se (blue). The corresponding PBDTfDTBX (X = O, T(S), Se) polymer fluorescence decays are shown in the inset.

To confirm the emissions from the CT states, we performed time correlated single photon counting (TCSPC) fluorescence decay measurements. Such emissions have been previously interpreted due to charge transfer states, where the long and weak emissive decay is attributed to CT emission^16^. Figure SI4 shows the steady-state fluorescence spectra of the neat polymers and their blends; fullerene quenches the fluorescence emission of the polymers and produces a new band peaking at 760 nm for all three blends. Figure 4 shows the TCSPC fluorescence decay measurements, which confirm that charge transfer emissions occur at a detection wavelength of 760 nm for the blends and at 690 nm for the neat polymers. The decay plots shown in Figure 4 for the polymers and blends are satisfactorily fitted bi-exponentially. The PBDTfDTBT and PBDTfDTBSe neat polymers show bi-exponential decay with a dominating short life time of 0.10 ns, and a weak longer life time of 0.96 ns, 1.1 ns respectively, as given in Table SI3. The fluorescence decays of blends are slower than those of the neat polymers. The fluorescence emissions of the PBDTfDTBT:PCBM and PBDTfDTBSe:PCBM blends also exhibit bi-exponential decay with weaker components of 2.1 ns and 4.6 ns respectively, that can be assigned to CT emissions. It is seen that fluorescence is quenched at longer time in PBDTfDTBSe:PCBM blend confirming the distribution of charge carriers in the CT states is twice as higher as PBDTfDTBT:PCBM blend. As mentioned earlier, this effect might be due to the larger size and lower electronegativity of the Se atom having its ease of interaction with the neighboring PCBM domains which also mean that its charge transfer time is slightly better than that of the PBDTfDTBT:PCBM blend. Nevertheless, PBDTfDTBT:PCBM blend produces only a very weak CT emission, which confirms that there are no other losses during the transfer of charge to the PCBM domains.

The dynamics of the PBDTfDTBO:PCBM blend is slightly different from those of the PBDTfDTBT:PCBM and PBDTfDTBSe:PCBM blends. Most of the PBDTfDTBO blend excited CT states are prone to strong fluorescence quenching, as indicated by fluorescence decay measurements. Further, the lifetime of its fluorescence TCSPC decay is the slowest of the blends, approximately 11 ns, as shown in Table SI3. For comparison, the fluorescence decay of the PBDTfDTBO polymer is also shown in Figure 4. However, the loss of charges during charge transfer to PCBM is higher in the PBDTfDTBO:PCBM blend than in the PBDTfDTBT:PCBM and, PBDTfDTBSe:PCBM blends. Thus, the long-lived emission of the PBDTfDTBO:PCBM blend indicates that the majority of the charges in the high or low lying CT states, decay and are quenched even with efficient charge transfer.

## Conclusions

We have investigated the influence of chalcogen heteroatoms in polymer solar cell materials on their charge carrier generation, charge transfer and recombination dynamics. Of the PBDTfDTBO:PCBM, PBDTfDTBT:PCBM, and PBDTfDTBSe:PCBM blends, the PBDTfDTBO:PCBM blend is dominated by nongeminate recombination and charge transfer losses. Instead, the PBDTfDTBT:PCBM blend exhibits charge transportation along with a minimum recombination losses in the carrier dynamics. Thus, PBDTfDTBT:PCBM blend containing S heteroatom turns out to be the perfect candidate for development of solar cells.

## Methods

### Materials

The medium-bandgap polymers PBDTfDTBO, PBDTfDTBT, PBDTfDTBSe were synthesized as described elsewhere^12^. Polymer/fullerene blend films were prepared on glass substrates by spin-coating from a chlorobenzene solution with PCBM of PBDTfDTBO, PBDTfDTBT, PBDTfDTBSe at a spin rate of 1500 rpm per 60 s under ambient conditions and annealead at 65 °C for 10 minutes. The blend solutions were stirred at 50 °C overnight to achieve homogeneity. The film thickness was typically 100–120 nm.

### Transient Absorption Measurements

Pump–probe differential transmission measurements were performed on the solar cell blend films at the ambient temperature. The light source for excitation was the home-built optical parametric oscillator (OPO) laser. Details of the apparatus have been described previously^26^. Group velocity dispersion (GVD) of the fundamental output of the OPO was compensated by a pair of SF 10 prisms to provide transform-limited 100 fs pulses at a probe wavelength of 1150 nm. The repetition rate and pulse energy were adjusted to 500 kHz and 30 nJ, respectively to prevent photodamage. Pump pulses at 550 nm were generated from the second harmonic generation in a 3 mm thick lithium triborate (LBO) crystal. The pump pulse energy at 550 nm was approximately 1 nJ. GVD of the optics along the pump beam path was compensated by a pair of fused silica prisms. The probe beam at 1150 nm was spectrally resolved by a bandpass filters with a bandwidth of 5 nm, prior to the photodetector. The full width at half maximum (FWHM) of the instrument response function (IRF) was estimated by the cross correlation between the pump and the gate pulses to be 120 fs.

### Time-Correlated Single Photon Counting (TCSPC) Measurements

The time-correlated single photon counting (TCSPC) method was used to record the photoluminescence lifetime profiles of the neat PBDTfDTBT, PBDTfDTBSe and PBDTfDTBO polymers and of their PC[70]BM blends on glass substrate. The light source for excitation was the home-built OPO laser. The apparatus has been described previously^26^. The output of the home-built OPO running in the near infrared region was doubled to generate the excitation pulses at 550 nm. The repetition rate was 500 kHz. A singlet lens was used to focus the excitation pulse onto the sample and the fluorescence was collected with a parabolic mirror. The fluorescence was dispersed with a monochromator (SP300, Acton), and detected with a single photon detection module (id 100–50, id Quantique). The FWHM of the IRF was 60 ps. Magic angle detection was used to prevent the effects of polarization. All measurements were performed at ambient temperature.

## Electronic supplementary material

Supplementary Information
