# Supplementary material for: Excitation Intensity Dependent Carrier Dynamics of Chalcogen Heteroatoms in Medium-Bandgap Polymer Solar Cells
Source: Sci Rep. 2017 Apr 11;7:836. doi: 10.1038/s41598-017-00834-0 (PMC5429773; doi:10.1038/s41598-017-00834-0)
Supplement: Supplementary file 1 — Supplementary Information [file 41598_2017_834_MOESM1_ESM.doc]

Supporting Information

Excitation Intensity dependent Carrier Dynamics of Chalcogen heteroatoms in Medium Bandgap Polymer Solar Cells

Chandramouli Kulshreshtha^†^, Jiwon Son^†^, Torbjörn Pascher, Ji-Hee Kim,

Taiha Joo, Jaewon Lee, Mun Seok Jeong and Kilwon Cho*

**Figure SI1(a)**. Transient absorption spectrum for PBDTfDTBT:PC[70]BM (1:1.5) blend.

**Figure SI1(b)**. Transient absorption spectrum for PBDTfDTBSe:PC[70]BM (1:1) blend.

**Figure SI1(c).** Transient absorption spectrum for PBDTfDTBO:PC[70]BM (1:1) blend.

**Figure SI2(a)**. Comparative transient absorption kinetics for PBDTfDTBO:PC[70]BM (black), PBDTfDTBT:PC[70]BM (olive), and PBDTfDTBSe:PC[70]BM (blue) blends at their highest excitation fluence (ph/cm^2^/pulse). The excitation fluence was 3.6×10^14^ ph/cm^2^/pulse for PBDTfDTBO:PC[70]BM blend, and 2.8×10^14^ ph/cm^2^/pulse for PBDTfDTBT:PC[70]BM and PBDTfDTBSe:PC[70]BM blends.

**Figure SI2(b)**. Comparative transient absorption kinetics for PBDTfDTBO:PC[70]BM (black), PBDTfDTBT:PC[70]BM (olive), and PBDTfDTBSe:PC[70]BM (blue) blends at their lowest excitation fluence (ph/cm^2^/pulse). The excitation fluence was 3.69×10^13^ ph/cm^2^/pulse for PBDTfDTBO:PC[70]BM blend, and 9.2×10^12^ ph/cm^2^/pulse for PBDTfDTBT:PC[70]BM and PBDTfDTBSe:PC[70]BM blends.

**
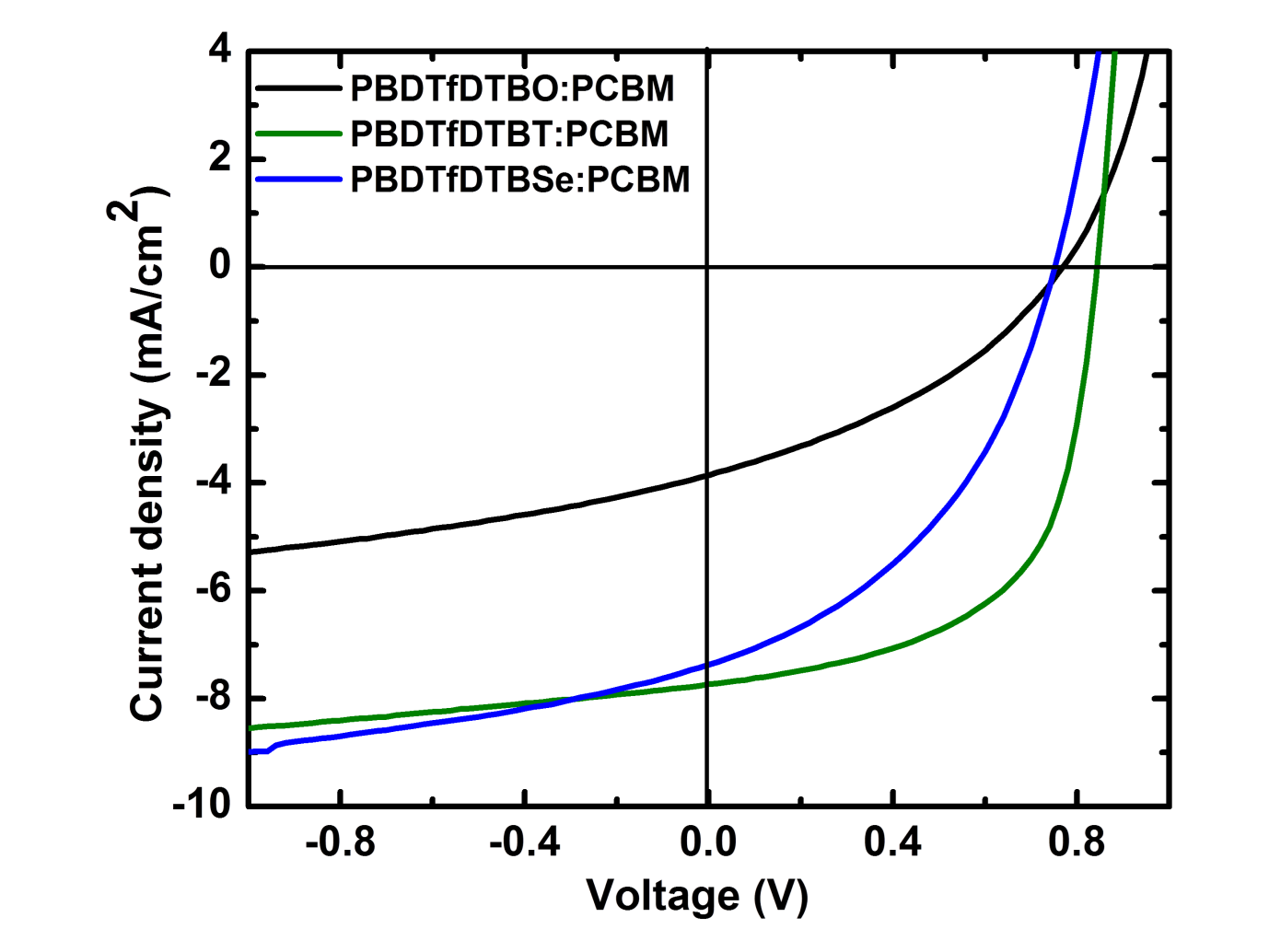
**

**Figure SI3**. Device characteristics of PBDTfDTBO:PC[70]BM, PBDTfDTBT:PC[70]BM, and PBDTfDTBSe:PC[70]BM blends.

**Figure SI4**. Steady-state spectrum for PBDTfDTBO (black), PBDTfDTBT (olive), and PBDTfDTBSe (blue) polymers (solid line), and their respective blends with PC[70]BM (dashed line).

Supporting information table

TableSI1. HOMO and LUMO energies of PC[70]BM and PBDTfDTBO, PBDTfDTBT, PBDTfDTBSe, polymers.

| **Polymers** | **HOMO (eV)** | **LUMO (eV)** | **MW (KDa)** |
| --- | --- | --- | --- |
| PBDTfDTBO | -5.5 | -3.4 | 18 |
| PBDTfDTBT | -5.4 | -3.4 | 89 |
| PBDTfDTBSe | -5.4 | -3.3 | 22 |
| PC[70]BM | -6.2 | -4.0 | - |

Table SI2. Solar cell device performance of PBDTfDTBO:PCBM, PBDTfDTBT:PCBM, and PBDTfDTBSe:PCBM.

|  | **PBDTfDTBO:PCBM** | **PBDTfDTBT:PCBM** | **PBDTfDTBSe:PCBM** |
| --- | --- | --- | --- |
| Blend ratios | 1:1 | 1:1.5 | 1:1 |
| Voc | 0.76 | 0.84 | 0.76 |
| Jsc | 3.86 | 7.74 | 7.37 |
| FF | 36.6 | 59.0 | 41.4 |
| PCE (%) | 1.07 | 3.84 | 2.32 |

Table SI3. Time correlated single photon counting decays with two exponential fits decay data for PBDTfDTBO:PCBM, PBDTfDTBT:PCBM, PBDTfDTBSe:PCBM blends, and their neat polymers.

|  | **t1(ns)** | **t2(ns)** |
| --- | --- | --- |
| PBDTfDTBO polymer | 0.11 | 1.0 |
| PBDTfDTBT polymer | 0.11 | 0.96 |
| PBDTfDTBSe polymer | 0.10 | 1.1 |
| PBDTfDTBO:PCBM | 0.82 | 11 |
| PBDTfDTBT:PCBM | 0.23 | 2.1 |
| PBDTfDTBSe:PCBM | 1.3 | 4.6 |
